# A Review on Plants and Microorganisms Mediated Synthesis of Silver Nanoparticles, Role of Plants Metabolites and Applications

**DOI:** 10.3390/ijerph19020674

**Published:** 2022-01-07

**Authors:** Tijjani Mustapha, Norashiqin Misni, Nur Raihana Ithnin, Abdullahi Muhammad Daskum, Ngah Zasmy Unyah

**Affiliations:** 1 Department of Medical Microbiology and Parasitology, Faculty of Medicine and Health Sciences, Universiti Putra Malaysia, Serdang 43400, Malaysia; tijjanimustapha@yahoo.com (T.M.); norashiqin@upm.edu.my (N.M.); raihana@upm.edu.my (N.R.I.); 2 Department of Biological Sciences, Faculty of Science, Yobe State University, Damaturu 620242, Nigeria; daskum341@gmail.com

**Keywords:** silver nanoparticles, green synthesis, plant metabolites

## Abstract

Silver nanoparticles are one of the most extensively studied nanomaterials due to their high stability and low chemical reactivity in comparison to other metals. They are commonly synthesized using toxic chemical reducing agents which reduce metal ions into uncharged nanoparticles. However, in the last few decades, several efforts were made to develop green synthesis methods to avoid the use of hazardous materials. The natural biomolecules found in plants such as proteins/enzymes, amino acids, polysaccharides, alkaloids, alcoholic compounds, and vitamins are responsible for the formation of silver nanoparticles. The green synthesis of silver nanoparticles is an eco-friendly approach, which should be further explored for the potential of different plants to synthesize nanoparticles. In the present review we describe the green synthesis of nanoparticles using plants, bacteria, and fungi and the role of plant metabolites in the synthesis process. Moreover, the present review also describes some applications of silver nanoparticles in different aspects such as antimicrobial, biomedicine, mosquito control, environment and wastewater treatment, agricultural, food safety, and food packaging.

## 1. Introduction

Nanotechnology is one of the emergent cutting-edge technologies in a variety of different fields of science including biology, chemistry, and material science [1]. The continued utilization of nanotechnology for fabricating nano-scale products in research and development is growing [2,3]. Nanoparticles are small fragments which have a nano-scale dimension that ranges between 1–100 nm, with a very good thermal conductivity, catalytic reactivity, non-linear optical performance and chemical steadiness owing to their large surface area to volume ratio [4]. They are generally classified into different groups based on their sizes, shapes, and properties. The different groups include carbon-based nanoparticles, metal nanoparticles, ceramic nanoparticles, and polymeric nanoparticles and so many others [5]. The nanoparticles can be synthesized using several methods including chemical, physical, and biological methods. However, the chemical and physical methods are expensive, quite complicated, and potentially dangerous for the environment due to the toxic chemical compounds used as reducing agents [1,6].

In recent times, the biological method otherwise termed the green synthesis method has received increased attention due to the growing need to develop an environmentally benign technology in nanoparticle synthesis [7]. Due to its simple processes and cost effectiveness, both the research and industrial sectors are currently interested in green production of silver nanoparticles for use in many applications in biomedicine, the environment, and industries [8]. Although there has been reported findings on the green synthesis of silver nanoparticles using several plants and microorganisms, the potential of plants and microorganisms as biological materials for the synthesis of silver nanoparticles is still yet to be fully explored.

The aim of this review was to examine the available published articles from 2001–2021 related to plant- and microorganism-mediated synthesis of silver nanoparticles, and the role of plant metabolites and applications. This review attempts to provide the general overview of various approaches of silver nanoparticles synthesis using green synthesis methods and the role of plant metabolites in the synthesis. The applications of silver nanoparticles in different aspects such as antimicrobes, biomedicine, mosquito control, environment controls, waste-water treatment, agricultural, food safety and food packaging will also be discussed. Lastly, the review provides direction and suggestions for future studies.

## 2. Methods of Silver Nanoparticles Synthesis

**Figure 1:**
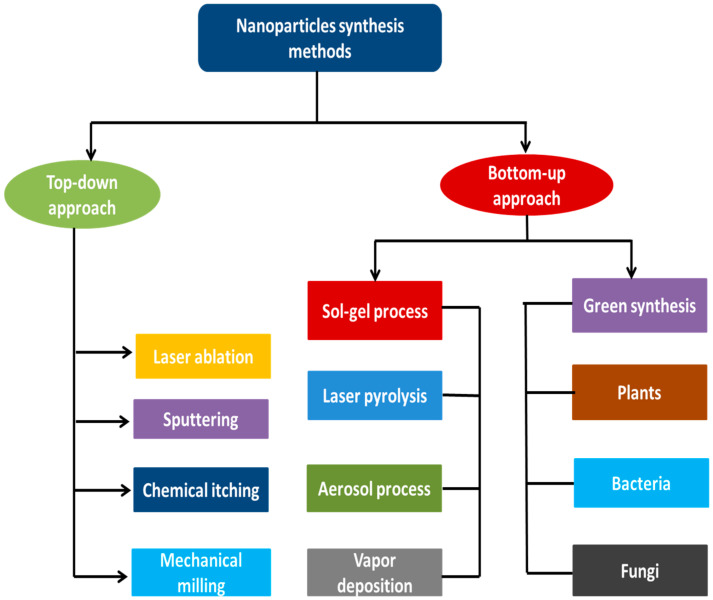
Different methods of synthesis of silver nanoparticles using the top-down and bottom-up approaches.

Silver nanoparticles (AgNPs) are synthesized using various physical, chemical, and biological techniques, which results in different shapes and sizes for use in numerous applications. These methods of synthesis are categorized into two main categories, namely “top-down” and “bottom-up” approaches. In the top-down approach, the size of silver metal in its bulk form is reduced mechanically to the nano-scale by using methods such as lithography, laser ablation, mechanical milling, etc. (Figure 1), whereas the bottom-up approach (self-assembly) involves the dissolution silver of salts in a solvent, reducing silver ions to their element with the use of a reducing agent, and then stabilizing the resulting neutral silver nanoparticles with stabilizing agents to prevent agglomeration [9,10]. A schematic diagram showing various top-down and bottom-up techniques for syntheses of silver nanoparticles is shown in Figure 1.

### 2.1. Top-Down Approach

In this method of synthesis, a destructive approach is used to produce silver nanoparticles. It begins with breaking down a larger molecule which is decomposed into smaller units and then these units are converted into suitable nanoparticles. It is energy-demanding and requires extensive processing. The major advantage of this technique is the control of the size distribution and morphologies of nanoparticles [11]. Laser ablation, lithography, grinding/milling, spray pyrolysis, evaporation-condensation, and other decomposition processes are examples of this approach [12]. These approaches (laser ablation, lithography, grinding/milling, spray pyrolysis, evaporation-condensation) are also regarded as the physical synthesis methods. During these processes, the foundation material is placed in the middle of a furnace and vaporized into a carrier gas. The evaporation-condensation method has already been used to make carbon, lead, gold and silver nanoparticles [13]. Furthermore, a typical tube furnace takes several minutes of pre-heating time to achieve a constant working temperature, as well as electricity of several kilowatts. One of the limitations in this technique is the defects in the surface structure of the product, whereas physical properties of nanoparticles are dependent on the structure of the surface. The top-down approach was employed to synthesize colloidal carbon spherical particles with controlled sizes. The synthesis process was based on the continuous adsorption of chemical polyoxometalates on the interfacial surface of the carbon. Adsorption made the carbon black aggregates into relatively smaller spherical particles, with high dispersion capacity and narrow size distribution [14].

### 2.2. Bottom-Up Approach

The bottom-up technique is the reverse of the top-down method; it involves bringing together atoms and molecules to produce a diverse range of nanoparticles, therefore this approach is also called the building-up approach. Examples of this approach include sol-gel process, laser pyrolysis, aerosol process, chemical vapor deposition, and biological agents-assisted synthesis [15]. In this approach, nanoparticles can be synthesized using either chemical or biological methods by the self-assembly phenomenon of atoms to new nuclei which grows into nano-scaled particles [16]. The chemical reduction method is one of the methods used in this approach to synthesized silver nanoparticles. Various organic and inorganic agents such polyethylene glycol, sodium citrate, N,N-dimethylformamide, ascorbate, and sodium borohydride are used for the reduction of silver ions [17]. The green and biological bottom-up synthesis method of nanoparticles has drawn more attention in recent times by many researchers due to their less toxic effect and feasibility of the approach. These processes are also eco-friendly and cost-effective. In this approach nanoparticle synthesis is accomplished using biological systems such as using plant extracts such as bacteria, yeast, and fungi [18].

## 3. Syntheses of Silver Nanoparticles Using Plant Extracts

Synthesis of silver nanoparticles using plant materials is an important branch of biosynthesis processes. It has been known for a long time that plants have the potential to reduce metal ions both on their surface and in various organs and tissues remote from the ion penetration site [19]. The bioactive molecules found in plants include enzymes, proteins, amino acids, vitamins, polysaccharides, and organic acids such as citrates are potentially able to reduce metal ions [20].

In this regard, plant extracts are used for the bio-reduction of the metal ions to form their nanoparticles. The extract of various parts of plants such as leaves, flowers, seeds, barks, fruits, and roots have been applied for synthesis of silver nanoparticles and the plant extracts may serve as stabilizing and reducing agents in the synthesis process [21,22,23]. Several studies have been carried out on the synthesis of silver nanoparticles using plant extracts. Gardea et al., 2003 [24] was first to report the synthesis of metallic nanoparticles using alfalfa sprouts. The alfalfa roots have the ability to absorb silver (Ag) from agar medium and voyage them into shoots of plant in same oxidation state. In shoots, these silver (Ag) atoms arranged themselves to produce silver nanoparticles (AgNPs). Ahmad and Sharma 2012 [6] also synthesized silver nanoparticles using *Ananas comosus* extract and characterized the synthesized silver nanoparticles using high resolution transmission electron microscopy (HRTEM), UV–Vis spectroscopy, energy dispersive X-ray spectroscopy (EDX), and selected area diffraction (SAD). The nanoparticles show a spherical shape with a diameter of 12 nm. In another study [25] silver nanoparticles were also synthesized using the leaf extract of *Argemone mexicana* that serve as reducing and capping agents. The properties of synthesized nanoparticles were analyzed using UV–Vis spectroscopy, X-ray diffraction (XRD), scanning electron microscopy (SEM), and Fourier transmission infrared spectrophotometer (FTIR). The average size of synthesized silver nanoparticles was 30 nm. Gavhane et al., 2012 [26] also synthesized silver by the reduction of aqueous AgNO_3_ solution using the extract of neem and triphala. The properties of nanoparticles were analyzed using EDX, nanoparticles tracking analysis (NTA), and transmission electron microscope (TEM). NTA and TEM revealed that the spherical particle sizes were 43 nm and 59 nm. Velmuurugun et al., 2015 [27] also reported the synthesis of silver nanoparticles using peanut shell extract and their characteristics showed a spherical and oval shape with an average diameter up to 10–50 nm. Roy et al., 2014 [28] synthesized nanoparticles using the extract of *Malus domestica* with an average diameter of 20 nm. The nanoparticles were analyzed using UV–Vis spectroscopy, and their distinctive phases and morphology were confirmed using XRD and TEM, and FTIR was used to identify the biomolecules which are responsible for the reduction and stabilization of nanoparticles. *Polyalthia longifolia* leaf extract was also used as a reducing agent to synthesize silver nanoparticles [29]. Silver nanoparticles were also synthesized by Maqdoom et al., 2013 [30] using the fruit extract of Papaya and were characterized using absorption spectroscopy and FTIR. Spherical-shaped silver nanoparticles were synthesized using *Ocimum sanctum* leaf extract and characterized using UV–Vis spectroscopy, SEM, and XRD [31]. A leaf extract of *Ceratonia siliqua* was used to synthesize spherical silver nanoparticles with average particle sizes between 5–40 nm. UV–Vis spectroscopy, atomic absorption spectroscopy (AAS), XRD, SEM, and FTIR were used to characterize the nanoparticles. *Jatropha curcas* leaf extract was also used in syntheses involving the reduction of aqueous silver nitrate (AgNO_3_) solution [21]. Silver nanoparticles were synthesized using the leaf extract of *Cassia auriculata,* and the leaf extract served as a reducing as well as a capping agent [32]. Shankar 2014 [33] synthesized silver nanoparticles using *Geranium* leaf extract. The silver nanoparticles synthesized were crystalline and stable with average size of size 40 nm. Stable and spherical shaped silver nanoparticles with average sizes between 10–50 nm were synthesized using the leaf extract of *Ficus benghalensis*. The characteristics of the synthesized nanoparticles were studied by FTIR, SEM, thermagravimetric analysis (TGA), and XRD [34]. Nakkala et al., 2014 [35] reported the syntheses of silver nanoparticles using *Acorus* *calamus* extract and evaluated its oxidation state, anti-cancer, and antibacterial effects. Another study by Kumar et al., 2014 [36] reported the syntheses of silver nanoparticles using the *Boerhaavia diffusa*. The synthesized nanoparticle showed an average size of 25 nm with a face-centered cubic geometry and spherical shape. The antimicrobial activity of synthesized silver nanoparticles was evaluated against *Pseudomonas fluorescens*, *Aeromonas hydrophila*, and *Flavobacterium*. Dwivedi et al., 2010 [37] also reported the syntheses of spherical shaped silver nanoparticles using the obnoxious weed *Chenopodium album*. The average nanoparticle sizes were between 10–30 nm as measured by TEM. In another study [38], Aldebasi et al. reported the synthesis of silver nanoparticles using *Ficus carica* leaf extract. Spherical shaped silver nanoparticles have been synthesized using the extract of *Abutilon indicum* and its antimicrobial activity was evaluated against *Salmonella typhi*, *Escherichia coli*, *Salmonella aureus*, and *Bacillus subtilis* microorganisms [39]. A similar study [40] by Awwad et al., reported on the syntheses of silver nanoparticles using the extract of Mulberry leaves. The 20 nm silver nanoparticles showed antimicrobial activity against *Staphylococcus aureus* and *Shigella* spp. Silver nanoparticles are synthesized by Awwad et al., 2012 [40] using the extract of *Olea europaea* and characterized by using SEM, XRD, and FTIR and yielded 15–40 nm particles sizes. Silver nanoparticles were synthesized by the reduction of silver nitrate (AgNO_3_) using the leaf extract of olive and the synthesized nanoparticles indicated effectiveness against drug-resistant bacteria isolates. The nanoparticles were characterized using UV–Vis spectroscopy, XRD, TGA, and SEM, and results showed that nanoparticles are mostly spherical with an average diameter between 20–25 nm [41]. *Alternanthera dentate* leaf extract was also used as a reducing and capping agent for synthesis of silver nanoparticles [42]. Studies by Murugan 2014 [43] reported that silver nanoparticles were synthesized using *Acacia leucophloea* extract with an average size that ranged between 38–72 nm. Another study by Arokiyaraj et al., 2014 [44] also reported that silver nanoparticles were synthesized using *Chrysanthemum indicum*, and nanoparticle sizes were 17–29 nm. Ashok et al., 2012 [45] and Kumar et al., 2013 [46] have synthesized silver nanoparticles using the leaf extract of *Parthenium hysterophorus*, *Premna herbacea*, and added to the aqueous solution of AgNO_3_. The detail information on the plant and plant extracts used for the synthesis of silver nanoparticles are given in Table 1.

The use of plant extract in green synthesis has been well studied (presented in Table 1) and studies are still ongoing. Studies have demonstrated that the formation of metal nanoparticles using plant extracts can be completed in the metal salt solution at short duration of time at room temperature depending upon the nature of the plant extract. The main factors that can affect the formation of the nanoparticles are the concentration of the extract, temperature, metal salt, pH, and contact time [47]. The importance of using plants in the synthesis of nanoparticles is that plants are easy to obtain and all parts of plant (roots, latex, stem, seeds, and leaves) possess a large number of active agents that can be used in the reduction of Ag ion [48]. The active agents present in the plants which are responsible for the possible reduction of silver ion to silver nanoparticles includes terpenoids, polysaccharides, phenolics, alkaloids, flavones, amino acids, alcoholic compounds enzymes, and proteins [49,50]. Therefore, plant extracts in green synthesis were found to be ideal candidates for synthesizing silver nanoparticles due to their rapid growth, non-pathogenic and eco-friendly reaction conditions that occur in a single step using a highly economical protocol.

## 4. Synthesis of Silver Nanoparticles Using Bacteria

Microorganisms such as bacteria, are of great interest for nanoparticle synthesis, although this process is facing challenges, such as culture contamination, lengthy procedures and less control over nanoparticles size [12]. The bacteria are known to possess the extraordinary ability of reducing heavy metal ions and are therefore regarded as one of the best candidates for the synthesis of nanoparticles. Studies have reported that some bacteria species like *Pseudomonas stutzeri* and *Pseudomonas aeruginosa* have a developing ability to resort to specific defense mechanisms to overcome stresses like toxicity of heavy metal ions or metals and even survive and grow despite high metal ion concentrations [56]. Several studies have reported the synthesis of silver nanoparticles using bacteria (Table 2). Klaus et al. [57] reported the synthesis of silver nanoparticles with well-defined compositions and shapes using the *Pseudomonas stutzeri* strain. This study was one of the earliest studies that synthesized silver nanoparticles using microorganisms. Shivaji and Madhu 2011, [58] synthesized silver nanoparticles using culture supernatants of psychrophilic bacteria. Nanda and Sravanan 2009 [59] also studied the synthesis of silver nanoparticles using *Staphylococcus aureus.* A study by Kalimuthu et al., 2008 [60] also reported the synthesis of silver nanoparticles using the biomass of *Bacillus licheniformis*. It was reported that the size of the nanoparticles synthesis was between 40–50 nm, and these were stabilized using an enzyme nitrate. The summary of different bacteria strains used in synthesis of silver nanoparticles are shown in Table 2.

## 5. Synthesis of Silver Nanoparticles Using Fungi

Fungi are one of the reducing and stabilizing agents used in the biosynthesis of silver nanoparticles. The fungi mediated synthesis of silver nanoparticles has attracted more attention because of its ability to produce commercial quantities of silver nanoparticles with controlled size, morphology, and low toxicity of residues [73]. In the synthesis of silver nanoparticles using microorganisms, fungi strains are preferred over bacteria species due to metal accumulation properties and better tolerance of the fungi [74,75]. The mechanism of synthesis of nanoparticles using fungi could be intracellular or extracellular. However, in the case of intracellular synthesis, the precursor metal is added to the mycelial culture and is co-opted in the fungal biomass. Consequently, the extraction of the nanoparticles is required after the synthesis using chemical treatment, centrifugation and filtration are employed to disrupt the biomass and release the nanoparticles [76]. In extracellular synthesis methods, the metal precursor is added to the aqueous filtrate that is made up of only the fungal biomass biomolecules, thereafter resulting in the formation of free nanoparticles. This method does not require any protocols to release the nanoparticles from the cells and is the most widely used method [77]. Balaji et al., 2009 [78] synthesized silver nanoparticles by extracellular synthesis methods using *Cladosporium cladosporioides.* The synthesized silver nanoparticles showed average sizes that range between 10–100 nm. Spherical shaped silver nanoparticles with an average size of 1–20 nm were synthesized using *Aspergillus terreus* [79]. Mukherjee et al., 2001 [80] also reported that mono-dispersed silver nanoparticles were synthesized by utilizing the fungus *Verticillium* by the green approach. The summary of different fungal strains used in syntheses of silver nanoparticles is shown in Table 3.

## 6. Plants Secondary Metabolites and Their Role in Synthesis of Nanoparticles

The crude extracts of different plants are known to contain wide ranges of primary and secondary metabolite compounds, ranging from proteins to different low molecular weight compounds such as phenolic acid, flavonoids alkaloid, terpenoids, amino acids, alcoholic compounds, glutathiones, polysaccharides, antioxidants, organic acids (ascorbic, oxalic, malic, tartaric, protocatechuic acid), and quinones. It is generally known that these metabolites are involved in redox reaction processes [93]. They are responsible for the reduction of metal ions into metallic nanoparticles. Although the elements participating in the green synthesis of nanoparticles and the underlying mechanism of the bio-reduction of ions is yet to be fully understood, it has been hypothesized that the bio-reduction of silver first involves the trapping of silver ions on the surface of proteins in the plant extract by means of electrostatic interactions [94]. The silver ions are then reduced by proteins, which lead to transformation of their secondary structure and the formation of silver nuclei. The silver nuclei later grow by further reduction of silver ions and their accumulation at the nuclei [95]. The involvement and participation of the secondary metabolites (sugars, terpenoids, polyphenols, alkaloids, phenolic acids, and proteins) in the reduction of metal ions leading to nanoparticle formation and in supporting their subsequent stability has also been postulated [19]. Based on available literature, flavonoid was one of the most frequently reported or predicted compounds responsible for the synthesis of silver nanoparticles in the green synthesis approach. Different plant species and plant metabolites responsible for the synthesis of silver nanoparticles reported in the literature are summarized in Table 4.

## 7. Applications of Silver Nanoparticles

**Figure 2:**
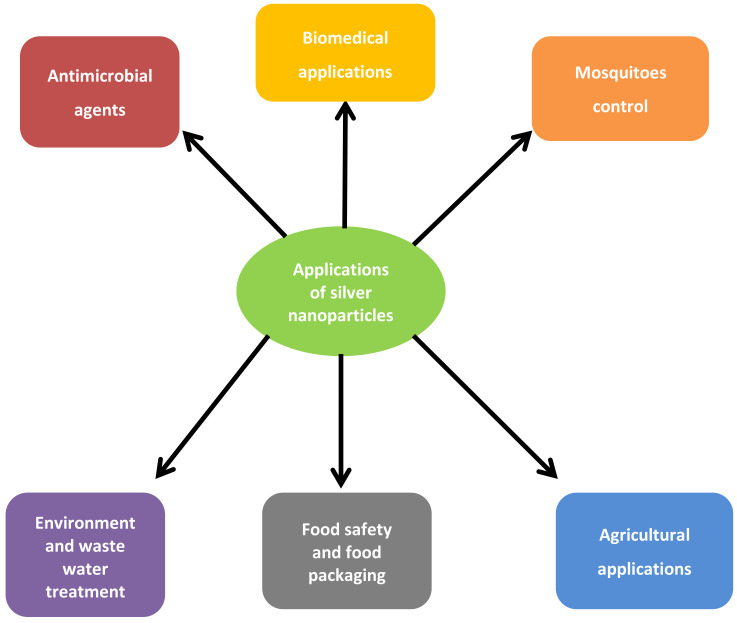
Applications of silver nanoparticles in different aspects.

Silver nanoparticles have been found to have a wide range of applications as antimicrobials in biomedicine, in environment and wastewater treatment, mosquito control, agriculture, food safety and food packaging, and so many other applications. A schematic diagram showing some applications of silver nanoparticles is shown in Figure 2.

### 7.1. Applications of Silver Nanoparticles as Antimicrobial Agents

Microorganisms are powerful and known to develop a resistance to different antibiotics. However, due to recent increases in bacterial resistance to various antibiotics, alternative therapeutic agents that are nontoxic to human beings but toxic to microorganisms are urgently required. Therefore, the development of nanoparticle mediated antimicrobial agents is most warranted. Recently, several studies have been focused on nanoparticle-based therapeutic agents against different bacteria species [114]. According to Ghaed et al., 2015 [115] silver nanoparticles have a strong toxicity to broad ranges of both gram negative and gram positive bacteria. However, the antimicrobial activity of silver nanoparticles (AgNPs) can be changed with physical properties such as size, shape, mass, and composition (pH, ions, and macromolecules) of the nanoparticle [22]. The shape of the nanoparticle can play a vital role in their antibacterial activity. For example, smaller silver nanoparticles may have greater binding surfaces and show more bactericidal activity compared to larger nanoparticles [116,117]. It has also been reported that the silver nanoparticles that range between 1–10 nm attach to the surface of the cell membrane and interact with the outer membrane of the bacteria, which arrest and disturb its proper functions such as respiration and permeability [118]. The antibacterial property of silver nanoparticles against *Staphylococcus aureus*, *Pseudomonas aeruginosa* and *Escherichia coli* has been investigated [119]. Recently, plant-based synthesized silver nanoparticles using *Lysiloma acapulcensis* have shown high antimicrobial potency against *E. coli*, *P. aeruginosa*, *S. aureus* and *C. albicans* compared to chemically produced silver nanoparticles. Furthermore, the study demonstrated that biogenic silver nanoparticles maintain lower cytotoxicity than the silver nanoparticles produced chemically [61].

### 7.2. Applications of Silver Nanoparticles in Biomedicine

The applications of silver nanoparticles in the field of biomedicine include drug delivery, cancer therapy, bio-imaging, dental technology, and many other applications. Recently, silver nanoparticles have attracted more attention in cancer therapeutics research because of their unique physical and chemical properties [120]. In comparison to available traditional anti-cancer therapies, metallic nanoparticles, especially silver nanoparticles, are considered as novel therapeutic agents or drug carriers in combination with drug candidates, and undesirable side-effects can be prevented by providing a targeted approach [121]. Previous studies have reported that silver nanoparticles exhibit good anti-cancer activities in different types of cancer, such as breast cancer, cervical, colon cancer, ovarian cancer, and lung cancer [122,123,124,125,126]. Furthermore, apart from anti-cancer therapy applications, nanoparticles have attracted more interest in the field of biomedicine because of their ability to deliver drugs in the optimum dosage range, often resulting in increased therapeutic efficiency of the drugs, weakened side effects and improved patient compliance [127]. Nanoparticles also have applications in cell bio-imaging and bio-sensing; however, the selection of the nanoparticles in question for achieving efficient contrast for cell imaging, biological application and photo thermal therapeutic applications is based on the optical properties of nanoparticles [128]. Silver nanoparticles also have applications in dentistry. It has been incorporated into some dental biomaterials for reducing biofilm formation due to its antibacterial activity. The silver nanoparticles joined into endodontic fillings showed a prolonged antibacterial impact against different strains of bacteria such as *Streptococcus milleri*, *Staphylococcus aureus* and *Enterococcus faecalis* [129]. The other applications of silver nanoparticles in biomedicine include wound dressings and catheters.

### 7.3. Applications of Nanoparticles in Environment and Waste Water Treatment

Metallic nanoparticles are one of the most attractive and cost-effective nanomaterials that have wide ranges of applications in the environment. The applications include environmental protection, water remediation, monitoring, purification, and treatment, and agriculture waste water treatment [3]. Clean and quality drinking water quality is one of the priorities of any nation. Plant extract-mediated green synthesized silver and gold nanoparticles can be employed for water treatment. For example, water and wastewater detoxification can be achieved by adsorption, photo-catalytic degradation, and nano-filtration techniques using nanoparticles as well [130]. Several studies have described the process of pesticides removal in water using gold and silver nanoparticles such as chlorprifos malathion and atrazine [131]. Moreover, the extraction of pesticides and other chemicals can be achieved by their adsorption onto nanoparticles which retain them on their surface, interacting for long periods until the complex precipitates. Therefore, these nanoparticles represent a suitable, convenient, and cost effective means of removing pesticides for either drinking water or irrigation projects. Water pollution with pathogenic microorganisms such as bacteria represent a high risk for water-borne diseases. The anti-microbial properties of metallic nanoparticles have been reported to be effective in this type of water purification [132].

### 7.4. Applications of Nanoparticles in the Control of Mosquitoes

The prevention and control of mosquitoes are important measures to control the spread of mosquito-borne diseases. The most commonly used method for the control of mosquitoes is chemical control. However, the persistent and repeated use of these synthetic chemical agents have caused the mosquito vector to become resistant, thus the need for the alternative new insecticides as novel biological tools for the control of mosquitoes [133]. Recently silver nanoparticles are emerging as one of the fastest growing materials due to their unique physical, chemical, and biological properties, small size and high specific surface area. Studies have indicated that synthesized silver nanoparticle using plants extracts of different plant species has shown to have tremendous mosquitocidal activity against various species of mosquitoes [133]. Moreover, nano-encapsulated pesticides and herbicides have shown enhanced properties in terms of solubility, specificity, permeability, and stability, as the nanostructure protects the active substance from early degradation and provides pest control for longer periods.

### 7.5. Applications of Nanoparticles in Agriculture

Nanotechnology in agriculture can provide wide ranges of applications for sustainable development through the development of nano-fertilizers, nano-pesticides, and nano-herbicides. Silver nanoparticle-based fertilizers have been developed in order to control nutrient release with plant uptake. This system helps to preserve or maintain soil fertility by reducing nutrient loss, contamination of ground water and soil, and chemical reactions between water, soil, and microbes that change them into unusable or dangerous chemicals for plants [134,135]. The control of pathogens such as bacteria and fungi responsible for causing disease in plants can be achieved by the spraying of nanoparticle solutions directly on seeds, grains, or foliage to prevent the invasion of plant pathogens. The nano-pesticides and nano-herbicides have shown enhanced properties in terms of solubility, stability, specificity, and permeability, as the nano-structure can protect and enhance the active substances from early degradation, and they provide active pest control ingredients for longer periods of time [3].

### 7.6. Applications of Nanoparticles in Food Safety and Food Packaging

The microbial contamination and spoilage of foods are major problems in food industries, considering the implication to public health due to food-borne diseases [136]. In order to minimize these problems, there is a need to develop active food packages with antimicrobial properties using active biocidal substances which may prevent food spoilage, microbial contamination, and increase the quality of the product. The materials used for packaging in food industries were organic acids, enzymes, and polymers (biodegradable and non-degradable). Recently, nanoparticles of metals or metallic oxides have been introduced with greater advantages compared with organic and inorganic acids, as they are resistant to the most severe processing conditions [136], such as exposure to high temperatures [137]. Therefore, the application of nanoparticles in the food packaging industry may offer potential solutions for the challenge presented by short shelf-life products, improving their quality and keeping them free of microbial adhesion. Metallic nanoparticles such as silver, magnesium oxide, copper oxide, zinc oxide, cadmium selenite/tellurite, titanium, and gold have been used in food industries due to their antimicrobial activity [138].

## 8. Conclusions

The green synthesis of silver nanoparticles offers a potentially ecofriendly, non-toxic, and cost-effective approach for the synthesis of nanoparticles. Different plant extracts and microorganisms can be used for the synthesis of silver nanoparticles. In the present review, the synthesis methods, applications, and role of plant metabolites in the synthesis of silver nanoparticles were highlighted. It is understood that different types of natural compounds present in plant extracts can act as reducing and stabilizing agents in the synthesis of silver nanoparticles. Plant-mediated silver nanoparticles are also stable due to the presence of natural capping agents such as proteins, which prevent the particles from aggregation. Furthermore, silver nanoparticles generated by green synthesis have potential applications, especially as antimicrobial agents of certain microorganisms for which their efficacy has been scientifically proven, in biomedicine as therapeutics agents, in mosquito control, in environment and wastewater treatment, in agriculture, in food safety, and in food packaging. Therefore, the green synthesis of silver nanoparticles using plant extracts has several advantages such as eco-friendliness, biocompatibility and cost-effectiveness. It is concluded that due to these unique properties, silver nanoparticles will have a key role in many of the nanotechnology-based processes.

## Figures and Tables

**Table 1:** Green synthesis of silver nanoparticles using plants.

Plant Species	Part of Plant Used	Size (nm)	Shape	References
*Nauclea latifolia*	Fruit	10	Irregular	[51]
*Citrus sinensis*	Peels	10–70	Spherical	[52]
*Cynara scolymus*	Leaf extract	98.47 ± 2.04	Spherical	[53]
Alfalfa sprouts	Plant shoot	2–3	Icosahedral	[24]
*Ananas comosus*	Plant broth	12	Spherical	[6]
*Argemone mexicana*	Leaves extract	30	Spherical	[25]
Neem and Triphala	Leaves extract	43–59	Spherical	[26]
Peanut	Shell extract	10–50	Spherical/oval	[27]
*Malus domestica*	Leaf extract	20	Spherical	[28]
*Polyalthia longifolia*	Leaf extract	35–10	Spherical	[29]
Papaya	Fruit extract	-	Spherical	[30]
*Ocimum sanctum*	Leaf extract	-	Spherical	[31]
*Ceratonia siliqua*	Leaf extract	5–40	-	[21]
*Cassia auriculata*	-	-	Spherical	[32]
*Geranium* spp.	Leaf extract	40	-	[33]
*Ficus benghalensis*	Leaf extract	10–50	Spherical	[34]
*Acorus calamus*	Rhizome	31.83	Spherical	[35]
*Boerhavia diffusa*	-	25	-	[36]
*Citrus limon*	Peel	59	Spherical	[54]
*Ananas comosus*	Fruit	5–30	Spherical	[6]
*Annona glabra*	Leaf extract	10–100	Spherical	[55]

**Table 2:** Synthesis of silver nanoparticles using bacteria species.

Bacteria Species	Size(nm)	Shape	References
*Escherichia coli*	1.2–62	Spherical/quasi-spherical	[61]
*Pseudomonas stutzeri*	200	Triangles and hexagons	[57]
*Serratia nematodiphila*	65–70	Spherical shape	[62]
*Bacillus stearothermophilus*	42–92	Spherical	[63]
*Lactobacillus casei*	25–50	Spherical	[64]
*Nocardiopsis* spp.	45 ± 0.15	Spherical	[65]
*Streptomyces hygroscopicus*	20–30	-	[66]
*Staphylococcus aureus*	160–180	Irregular	[59]
*Rhodococcus* spp.	5–50	Spherical	[67]
Marine *Ochrobactrum* spp.	38–85	Spherical	[68]
*Escherichia coli*	1–100	Spherical	[69]
*Lactobacillus* strains	15–500	Triangular/hexagonal	[70]
*Bacillus methylotrophicus*	10–30	Spherical	[71]
*Vibrio alginolyticus*	50–100	Crystalline/spherical	[62]
*Fusarium semitectum*	1–50	Ellipsoid/spherical	[72]

**Table 3:** Synthesis of silver nanoparticles using fungal species.

Fungi Species	Size (nm)	Shape	References
*Aspergillus niger*	1–20	Polydispersed spherical	[45]
*Alternaria alternata*	32	Spherical	[81]
*Penicillium fellutanum*	5–25	Spherical	[82]
*Fusarium semitectum*	10–60	Crystlline/spherical	[83]
*Schizophyllum commune*	51–93	Spherical	[84]
Endophytic fungus	10–25	Hexagonaerel/spherical	[85]
*Trichoderma viride*	5–40	Spherical	[86]
*Pestalotia* spp.	12	Polydispersed/spherical	[87]
*Penicillium citrinum*	109	Uniform spherical	[88]
*Fusarium acuminatum*	13	Spherical	[89]
*Aspergillus niger*	1–20	Polydispersed/spherical	[45]
*Fusarium oxysporum*	5–13	Spherical	[90]
*Guignardia mangiferae*	5–30	Spherical	[91]
*Duddingtonia flagrans*	30–409	Spherical	[77]
*Arthroderma fulvum*	21	Spherical	[92]

**Table 4:** Plant metabolites responsible for synthesis of silver nanoparticles in different plant species.

Plant Species	Metabolites Identified	References
*Acalypha indica*	Quercetin	[96]
*Nigella arvensis*	Flavonoids, alkaloids	[97]
*Lantana camara*	Flavonoids	[98]
*Mimusops elengi*	Polyphenols	[99]
*Zingiber officinale*	Flavonoid, alkaloids	[100]
*Solanum xanthocarpum*	Alkaloids, phenolic, sugars	[101]
*Trianthema decandra*	Saponin	[102]
*Aegle marmelos*	Tannin	[103]
*Anacardium occidentale*	Proteins, polyols	[104]
*Desmodium triflorum*	Ascorbic acid	[105]
*Decalepis hamiltonii*	Polyols, phenols	[106]
*Syzygium cumini*	Polyphenols	[107]
*Azadirachta indica*	Flavonoids, terpenoids	[108]
*Coleus aromaticus*	Flavonoids	[109]
*Hibiscus rosa-sinensis*	Carboxylate ion groups	[110]
*Helianthus annuus*	Flavonoids, proteins,	[98]
*Dioscorea bulbifera*	Flavonoids, polyphenols	[111]
*Glycyrrhiza glabra*	Flavonoids and terpenoid	[112]
*Achyranthes aspera*	Polyols	[113]

## Data Availability

Not applicable.
